# Postpartum-specific anxiety as a predictor of infant-feeding outcomes and perceptions of infant-feeding behaviours: new evidence for childbearing specific measures of mood

**DOI:** 10.1007/s00737-017-0775-0

**Published:** 2017-09-21

**Authors:** Victoria Fallon, Jason Christian Grovenor Halford, Kate Mary Bennett, Joanne Allison Harrold

**Affiliations:** Institute of Psychology, Health and Society, University of Liverpool, Eleanor Rathbone Building, Bedford Street South, Liverpool, L69 7ZA UK

**Keywords:** Postpartum anxiety, Postpartum-specific anxiety, Breastfeeding, Infant feeding behaviours, Psychometrics

## Abstract

Studies of pregnancy-specific anxiety suggest that it is a distinct construct which predicts perinatal outcomes more effectively than other general measures of anxiety. In response, a novel measure of postpartum-specific anxiety (PSAS) has been developed and validated, but it is not yet clear whether it possesses the same predictive power as its pregnancy-specific counterparts. The aim of this short-term prospective study was to (a) test the predictive validity of the PSAS in the context of one specific perinatal outcome, infant-feeding, and (b) examine whether the PSAS may be more efficacious at predicting infant-feeding outcomes and behaviours than the more commonly used general measures. Eight hundred mothers of infants aged between 0 and 6 months completed the PSAS alongside general measures of anxiety and depression at baseline. A subsample (*n* = 261) returned to complete a follow-up questionnaire examining infant-feeding outcomes and behaviours two weeks later. Hierarchical regression models revealed that the PSAS was associated with lower odds of breastfeeding exclusively, and breastfeeding in any quantity in the first 6 months postpartum. PSAS scores were also significantly associated with infant-feeding behaviours including a lower perceived enjoyment of food, and greater perceived food responsiveness and satiety responsiveness in the infant. As hypothesised, the PSAS was a stronger predictor of infant-feeding outcomes and behaviours than general anxiety and depression. The findings provide evidence for the predictive validity of the PSAS and call for the use of childbearing specific measures of mood when attempting to predict perinatal outcomes. Replication of these findings across other indices of maternal and infant health is now necessary.

## Introduction

Postpartum anxiety (PPA) remains among the most under-studied, under-diagnosed, and under-treated complications of childbirth (Smith and Kipnis 2012). Moreover, PPA has been associated with a variety of suboptimal outcomes in the infant (Lonstein 2007; Glasheen et al. 2010). Research into PPA currently utilises a range of general self-report measures which are seldom validated for use postpartum. This may lead to erroneous data, inaccurate interpretation, and incomparable results across studies (Meades and Ayers 2011).

These oversights have been addressed in the pregnancy anxiety literature (Van den Bergh 1990; Levin 1991; Wadwha et al. 1993; Huizink et al. 2002) where a distinct presentation from general anxiety and depression (Huizink et al. 2004) has been revealed. As a result, a number of self-report measures have been developed (e.g. Pregnancy Related Anxiety Questionnaire (PRAQ; Van Den Bergh 1990), and the PRAQ-R (Huizink et al. 2004)). Studies using these scales consistently find that pregnancy-specific anxiety is a more efficacious predictor of perinatal outcomes than other general forms of stress, anxiety, and depression (Guardino and Schetter 2014). This is evident across studies of pre-term birth (Dunkel Schetter 2011), cognitive and motor performance (Davis and Sandman 2010), attention regulation (Huizink et al. 2002), temperament (Davis et al. 2004), and infant-feeding (Fairlee et al. 2009).

An equivalent measure of postpartum-specific anxiety has recently been developed and validated (Fallon et al. 2016b). The Postpartum-specific Anxiety Scale (PSAS) is a 51-item scale that taps into four domains of anxiety which are specific to the postpartum period. The PSAS proved acceptable to postpartum women and demonstrated high validity and reliability in initial psychometric work. As with all novel measures, validation is an iterative process and the predictive utility of the PSAS has not yet been examined in comparison to its pregnancy-specific counterparts.

One fundamental infant health outcome lies in the nourishment of the infant. Appropriate infant-feeding (i.e. responsive maternal feeding, exclusive breastfeeding to 6 months of age) confers significant health benefits. A recent systematic review provides evidence that women with PPA are less likely to breastfeed exclusively, and more likely to terminate breastfeeding earlier (Fallon et al. 2016a). Furthermore, mothers who report anxiety are at risk of non-responsive feeding behaviours (Hurley et al. 2008) characterised by impaired feeding interactions, insensitivity to infant cues of hunger and satiety, and lack of uptake to current feeding recommendations (Birch and Fisher 1995; Hughes et al. 2005).

The aims of this paper are twofold. Firstly, the predictive validity of the PSAS will be examined within the context of infant-feeding using a short-term prospective design. Second, it will be examined whether the PSAS may be more efficacious at predicting infant-feeding and perceptions of infant-feeding behaviours than the more commonly used general measures. It is hypothesised that after controlling for the effects of general anxiety and depression, postpartum-specific anxiety will have a significant, independent effect on infant-feeding outcomes and perceptions of infant-feeding behaviours.

## Methods

### Participants

Mothers of infants aged between birth and 6 months postpartum were recruited via online advertising techniques providing a link to the Qualtrics survey software. A self-selecting subsample returned to complete the follow-up questionnaire 2 weeks later.

### Design and procedure

A short-term online prospective design was utilised. The main questionnaire comprised the maternal mental health measures (i.e. PSAS, STAI-S, STAI-T, BDI-II) in addition to demographic variables. This was accessible from 4 September 2015 to 5 November 2015.

Participants were then asked if they would like to return 2 weeks later to complete a follow-up survey. Those who were willing received an e-mail with the follow-up survey (BEBQ, feeding outcome items) exactly 2 weeks later. The link to the follow-up questionnaire was only active on the day it was distributed. Participants completing both questionnaires received a reimbursement of £10.

### Measures

#### Demographics

Maternal and infant demographic questions were asked at the beginning of the main questionnaire (see Table 1). Maternal height and weight values were converted to metric units and a maternal BMI (kg/m^2^) variable was computed for analyses. Occupational prestige, educational attainment, size of household, and living status were combined to create a composite measure of socio-economic status (SES) for analyses. Infant weight and length values were converted to metric units and infant BMI z-scores and percentiles were calculated using weight, length, age, and gender information.

**Table 1:** Maternal and infant characteristics (*N* = 261)

Maternal characteristic	Value	Infant characteristic	Value
Maternal age (mean years ± SD)	31.25 (± 4.50)	Infant age (mean weeks ± SD)	16.10 (± 6.43)
Country of residence (*N*/%)		Gender (*N*/%)	
UK	239 (91.6)	Male	146 (55.9)
Ireland	4 (1.5)	Female	115 (44.1)
USA	4 (1.5)	Birth order (*N*/%)	
Australia and NZ	2 (0.8)	1st	121 (46.4)
Other European	9 (3.4)	2nd	104 (39.8)
Other non-European	3 (1.2)	3rd	27 (10.3)
Marital status (*N*/%)		4th	4 (1.5)
Married	195 (74.7)	5th and after	5 (1.9)
Co-habiting	57 (21.8)	Birth weight (mean kg ± SD)	3.50 (0.69)
Single	7 (2.7)	Infant BMI percentile (mean ± SD)	30.80 (37.0)
Separated/divorced/widowed	2 (0.8)	Timing of birth (*N*/%)	
Occupation (*N*/%)		Premature (< 37 weeks)	7 (2.7)
Managers, directors, and senior officials	6 (2.3)	Early term (> 37, < 39 weeks)	49 (18.7)
Professionals	34 (13.0)	Full term (> 39, < 41 weeks)	124 (47.5)
Skilled trades	23 (8.8)	Late term (>41, < 42 weeks)	77 (29.5)
Caring, leisure, and other service	22 (8.4)	Post term (> 42 weeks)	4 (1.5)
Sales and customer service	2 (0.8)	Multiple birth (*N*/%)	
Process, plant, and machine operatives	31 (11.9)	Yes	4 (1.5)
Elementary occupations	6 (2.3)	No	257 (98.5)
Housewife	116 (44.4)		
Not in paid occupation	21 (8.0)	Infant feeding outcomes and behaviours	Value
Educational attainment (*N*/%)		EBF (*N*/%)	
Postgraduate education	64 (25.2)	Yes	176 (67.4)
Undergraduate education	123 (46.6)	No	85(32.6)
A-levels or equivalent college education	50 (18.9)	Any BF (*N*/%)	
GCSEs or equivalent secondary school education	16 (6.1)	Yes	217 (83.1)
Other qualification	7 (3.8)	No	44 (16.9)
No qualifications	1 (0.4)	EBF intention (*N*/%)	
Living status (*N*/%)		Yes	210 (80.4)
Own property	180 (68.9)	No	51 (19.6)
Rent privately	59 (22.6)	Any BF intention (*N*/%)	
Rent from the authority	11 (4.2)	Yes	253 (97.0)
Live with parents	2 (0.9)	No	8 (3.0)
Other	9 (3.4)	Timing of ICF (*N*/%)	
Size of household (inc. participant) (*N*/%)		< 6 months	53 (20.3)
2 people	7 (2.7)	6 months or after	208 (79.7)
3 people	113 (43.3)	Enjoyment of food (mean ± SD)^a^	4.20 (± 0.69)
4 people	102 (39.1)	Food responsiveness (mean ± SD)^a^	2.43 (± 0.78)
5 people	29 (11.1)	Satiety responsiveness (mean ± SD)^a^	2.27 (± 0.74)
6 or more people	10 (3.8)	Slowness in eating (mean ± SD)^a^	2.68 (± 0.82)
Current diagnosis of anxiety/depression (*N*/%)		General appetite (mean ± SD)^a^	3.79 (± 0.99)
Yes	27 (10.3)		
No	233 (89.3)		
Prefer not to say	1 (0.4)		
Maternal BMI (kg/m^2^) (mean ± SD)	27.00 (6.69)		

*EBF* exclusive breastfeeding, *BF* breastfeeding, *ICF* introduction to complementary feeding

^a^BEBQ Infant Feeding Behaviour Scores range between 1 and 5 with higher scores indicating higher perceived levels of each feeding behaviour

#### The Postpartum-specific Anxiety Scale (PSAS; Fallon et al. 2016b)

The PSAS (see Fallon et al. 2016b for items and factor loadings) is designed to measure the frequency of maternal and infant-focused anxieties experienced during the past week. It contains 51 items across four distinct constructs specific to the first 6 months after birth. ‘Competence and attachment anxieties’ (15 items) addresses anxieties relating to maternal self-efficacy, parenting competence and the mother-infant relationship. ‘Safety and welfare anxieties’ (11 items) examines fears about infant illnesses, accidents, and cot death. ‘Practical baby care anxieties’ (7 items) covers anxieties that are specific to infant care such as feeding, sleeping, and general routine. ‘Psychosocial adjustment to motherhood’ (18 items) addresses postpartum adjustment concerns including management of personal appearance, relationships and support, work and finances, and sleep. The PSAS was found to be acceptable to postpartum women and performed well in reliability and validity analyses.

#### The Beck Depression Inventory-II (BDI-II; Beck et al. 1988)

The BDI is a commonly used self-report tool for detecting and measuring general depression. It contains 21 items designed to measure the severity of general depression experienced during the past 2 weeks. Higher scores indicate more severe depressive symptoms. Twenty-five years of psychometric testing provides evidence of its reliability and validity in clinical and non-clinical samples (Beck et al. 1988).

#### The Spielberger State-Trait Anxiety Inventory (STAI; Spielberger et al. 1970)

The STAI is a self-report measure designed to capture levels of general anxiety. It contains 40 items with two separate subscales (20 items each) to measure situational (state) and stable (trait) anxiety. Higher scores on each four-point Likert scale item indicate higher levels of anxiety. The STAI is a reliable and valid measure used with clinical and non-clinical populations and more recently in perinatal samples (Meades and Ayers 2011; Spielberger et al. 1970).

#### Infant-feeding outcomes

Two researcher-developed 7-point Likert-scale items were used to ascertain current feeding method and prenatal feeding intention. Available answers were based on WHO-defined categories (WHO 2002). Mothers were asked ‘How are you currently feeding your baby?’ and available response options were as follows: ‘exclusively breastfeeding (100%)’, ‘predominately breast milk (over 80%) with a little formula (under 20%)’, ‘mainly breast milk (50%–80%) with some formula’, ‘a combination of both breast milk (50%) and formula (50%)’, ‘mainly formula (50%–80%) with some breast milk’, ‘predominately formula (over 80%) with a little breast milk (under 20%)’, and ‘exclusively formula feeding (100%)’. Mothers were then asked ‘How were you planning to feed your baby in pregnancy’, and the same response options were provided.

#### Baby Eating Behaviour Questionnaire (BEBQ; Llewellyn et al. 2011)

The BEBQ is a 17-item parental-report measure of infant-feeding behaviour during the period of exclusive milk feeding. It comprises four distinct feeding traits and one item describing general appetite. ‘Enjoyment of food’ (4 items) describes the infant’s perceived liking of milk and of feeding in general. ‘Food responsiveness’ (6 items) relates to how demanding the infant is with regard to being fed and his or her level of responsiveness to cues of milk and feeding. ‘Slowness in eating’ (4 items) evaluates the speed with which an infant typically feeds, and ‘satiety responsiveness’ (3 items) examines how easily the infant gets full during a feed. The item ‘My baby has a big appetite’ correlated with all scales and can be used as an individual item to measure overall appetite. The BEBQ demonstrated good reliability and validity in initial psychometric testing (Llewellyn et al. 2011).

### Method of analysis

To develop a comprehensive model, a range of potentially confounding variables was identified from previous literature (see Table 1). Bivariate analyses were conducted between each potential confounder, the exposure of interest (i.e. PSAS scores), and the outcome of interest (i.e. feeding outcome or behaviour). Confounders significantly associated with both exposure and outcome at 10% level were included in the final regression models.

The current feeding method categories (*N* = 5) were collapsed into two binary variables: (‘exclusively breast feeding’ yes/no, and ‘any breastfeeding’ yes/no). Concurrently, the initial feeding intention categories were also collapsed (‘exclusive breastfeeding intention’ yes/no, and ‘any breastfeeding intention’ yes/no). Two hierarchical binary logistic regressions (HLRs) were conducted to analyse the effect of PSAS scores in the main questionnaire on infant-feeding outcomes in the follow-up questionnaire. Relevant confounders were entered in block 1, followed by general measures of anxiety and depression in block 2. The PSAS was entered into the final block. Odds ratios (ORs) and 95% confidence intervals (CIs) were calculated to describe the predictive value of each variable.

Using the same entry method, a hierarchical linear multiple regression analysis (HMR) was conducted to analyse the effect of PSAS scores in the main questionnaire on perceptions of infant-feeding behaviours at follow-up. *β* and *p* values were calculated to describe the predictive value of each variable. Variance inflation factors (VIFs) were > 5 for the general measures of anxiety and depression in block 2 which warrants concern. The three measures (STAI-S, STAI-T, BDI) were converted to z-scores and combined, and the regression was conducted again with the composite variable. Results (*R* ^2^, *β*, and *p* values) were analogous, so the original entry method was used to provide the most informative output.

## Results

### Participants

Of the 1282 recruited, a total of 800 (62%) completed the main questionnaire. Of these, 261 returned to complete the follow-up questionnaire (33%). Among those completing both surveys, maternal age ranged from 19 to 44 years (M = 31.25; SD = 4.50). The sample were predominately married (75%), primiparous (46%), and housewives (44%) from the UK (92%). Twenty-seven (10%) of the women had a current, clinical diagnosis of anxiety or depression which is comparable with UK prevalence estimates. The infant age ranged from 1 to 26 weeks (M = 16.10; SD = 6.43). Sixty-seven percent of the infants were exclusively breastfed, and 83% of the infants were receiving breastmilk in any quantity. See Table 1 for demographic details. There were no difference in mean scores on any of the mood measures between those completing both surveys and those completing only the first survey (PSAS: *t* = 0.86, *p* = .39; STAI-S: *t* = 1.28, *p* = .20; STAI-T: *t* = 1.30, *p* = .19; BDI: *t* = 0.02, *p* = .99). Mothers completing both surveys did not differ from those completing only the first survey with respect to age, marital status, and BMI. However, mothers completing both surveys were more likely to have higher SES scores (20.85 ± 3.33 vs 20.23 ± 3.59; *t* = −2.38, *p* = .02) and less likely to have a current, clinical diagnosis of anxiety or depression (22.1 vs 77.9%, *χ* ^2^ = 4.57, *p* = .03). Infants of mothers completing both surveys did not differ from those only completing the first survey on any characteristic.

### HLR predicting exclusive breastfeeding status (Table 2)

The final regression model significantly predicted exclusive breastfeeding status, correctly identifying 79.9% of cases: Cox and Snell *R* ^2^ = .24, Nagelkerke *R* ^2^ = .33, *p <* .001. The covariates in step 1 explained approximately 20% (Cox and Snell) and 29% (Nagelkerke) of the variance in exclusive breastfeeding. General measures of anxiety and depression (step 2) explained approximately 2% (Cox and Snell) and 2% (Nagelkerke) of the variance but were not significant predictors of exclusive breastfeeding. However, in the final step, the PSAS was a significant predictor of exclusive breastfeeding which explained approximately 2% (Cox and Snell) and 2% (Nagelkerke) of the variance. Higher PSAS scores were associated with lower odds of exclusive breastfeeding (OR 0.98; CI 0.96, 0.97).

### HLR predicting any breastfeeding status (Table 2)

The final regression model significantly predicted any breastfeeding status, correctly identifying 85.6% of cases: Cox and Snell *R* ^2^ = .17, Nagelkerke *R* ^2^ = .29, *p <* .001. The covariates in step 1 explained approximately 14% (Cox and Snell) and 23% (Nagelkerke) of the variance in any breastfeeding. General measures of anxiety and depression (step 2) explained approximately 2% (Cox and Snell) and 3% (Nagelkerke) of the variance but were not significant predictors of any breastfeeding. However, in the final step, the PSAS was a significant predictor of exclusive breastfeeding which explained approximately 1% (Cox and Snell) and 3% (Nagelkerke) of the variance. Higher PSAS scores were associated with lower odds of any breastfeeding (OR 0.97; CI 0.95, 0.99).

**Table 2:** Hierarchical logistic regression demonstrating postpartum specific anxiety as a predictor of exclusive breastfeeding status and any breastfeeding status after controlling for general measures of mood

Variables	Step 1			Step 2			Step 3		
	*B*(SE)	OR	95% CI	*B*(SE)	OR	95% CI	*B*(SE)	OR	95% CI
Exclusive breastfeeding (yes/no)									
Step 1									
Timing of ICF	*0.77 (0.23)*	*2.16*	*1.38–3.37*	*0.77 (0.23)*	*2.15*	*1.36–3.40*	*0.73 (0.24)*	*2.07*	*1.30–3.29*
EBF intention									
Yes (1)	*2.22 (0.40)*	*9.21*	*4.22–20.13*	*2.26 (0.41)*	*9.57*	*4.32–21.20*	*2.33 (0.42)*	*10.28*	*4.55–23.28*
No (0)	–	–	–	–	–	–	–	–	–
Any BF intention									
Yes (1)	0.87 (1.14)	2.38	0.25–22.34	0.82 (1.15)	2.28	0.24–21.71	0.93 (1.16)	2.52	0.26–24.39
No (0)	–	–	–	–	–	–	–	–	–
Step 2									
BDI				0.01 (0.03)	1.01	0.95–1.07	0.03 (0.03)	1.03	0.96–1.10
STAI-S				− .05 (0.03)	0.95	0.90–1.00	− 0.04	0.96	0.91–1.01
STAI-T				0.03 (0.03)	1.03	0.98–1.08	0.04	1.04	0.99–1.09
Step 3									
PSAS							*− 0.03 (0.01)*	*0.98*	*0.96–0.097*
Any breastfeeding (yes/no)									
Step 1									
Timing of ICF	*0.56 (0.24)*	*1.76*	*1.09–2.82*	*0.55 (0.25)*	*1.73*	*1.06–2.85*	0.51 (0.26)	1.66	0.99–2.76
Maternal age	0.07 (0.04)	1.07	0.99–1.16	0.08 (0.04)	1.08	1.00–1.17	0.06 (0.04)	1.07	0.98–1.16
EBF intention									
Yes (1)	*1.69 (0.40)*	*5.41*	*2.46–11.91*	*1.75 (0.41)*	*5.73*	*2.56–12.83*	*1.78 (0.42)*	*5.95*	*2.62–13.52*
No (0)	–	–	–	–	–	–	–	–	–
Any BF intention									
Yes (1)	1.38 (0.91)	3.99	0.68–23.56	1.33 (0.92)	3.79	0.63–22.89	1.51 (0.93)	4.53	0.74–27.77
No (0)	–	–	–	–	–	–	–	–	–
Step 2									
BDI				0.01 (0.04)	1.01	0.94–1.08	0.04 (0.04)	1.04	0.96–1.12
STAI-S				− 0.06 (0.03)	0.94	0.89–1.00	− 0.05 (0.03)	0.96	0.90–1.02
STAI-T				0.03 (0.03)	1.03	0.97–1.09	0.04 (0.03)	1.04	0.98–1.11
Step 3									
PSAS							*− 0.03 (0.01)*	*0.97*	*0.95–0.99*

*﻿EBF﻿:R* ^2^ (block 3) = .24 (Cox and Snell); .33 (Nagelkerke). Step 1 block *χ* ^2^ = 60.21, *df* = 3, *p* < .001. Step 2 block *χ* ^2^ = 5.57, *df* = 3, *p* = .14. Step 3 block *χ* ^2^ = 5.60, *df* = 1, *p* = .018. Any BF: R2 (block 3) = .17 (Cox & Snell); .29 (Nagelkerke). Step 1 block χ2 = 39.61, df = 4, p<.001. Step 2 block χ2 = 5.75, df = 3, p=.13. Step 3 block χ2 = 5.07, df = 1, p=.02. Significant (*p* < .05) odds ratios (ORs) are indicated in italics

*SE* standard error, *CI* confidence interval, *EBF* exclusive breastfeeding, *BF* breastfeeding, *ICF* introduction to complementary feeding

### HMR predicting infant enjoyment of food (Table 3)

The final regression model predicted approximately 21% of the variance in general appetite scores (*R* ^2^ = .21, *F* (7,252) = 9.28, *p* < .001). The covariates in step 1 explained approximately 8% of the variance in enjoyment of food but were not significant predictors. General anxiety and depression (step 2) explained approximately 9% of the variance; again, these predictors were not significant. However, in the final step, the PSAS was a highly significant predictor which explained approximately 4% variance in enjoyment of food. Higher PSAS scores were associated with lower perceived enjoyment of food in the infant (*β* = − 0.33; *p <* .001).

**Table 3:** Hierarchical regression analysis demonstrating postpartum-specific anxiety as a predictor of infant enjoyment of food after controlling for general measures of mood

Enjoyment of food	Cumulative		Simultaneous	
	*R* ^2^-change	*F* ***-***change	*β*	*p*
Step 1				
Anxiety/depression diagnosis	.08	*F* (3,256) = 7.21**	− 0.03	.62
EBF			0.04	.52
Any BF activity			0.08	.30
Step 2				
BDI	.09	*F* (3,253) = 8.98**	− 0.05	.71
STAI-S			− 0.02	.86
STAI-T			− 0.01	.94
Step 3				
PSAS	.04	*F* (1,252) = 12.12**	*− 0.33*	*<.001*

Also after controlling for covariates identified as significant confounders in bivariate analyses in step 1. Entries in italics indicate significant *β* and *p* values

*EBF* exclusive breastfeeding, *BF* breastfeeding

* *p < .001

### HMR predicting infant food responsiveness (Table 4)

The final regression model predicted approximately 27% of the variance in food responsiveness scores (*R* ^2^ = .27, *F* (8,251) = 11.71, *p* < .001). The covariates in step 1 explained approximately 12% of the variance in food responsiveness. General anxiety and depression scores (step 2) explained approximately 9% of the variance in scores, although these predictors were not significant. In the final step, the PSAS was a highly significant predictor which explained approximately 6% of the variance in food responsiveness scores. Higher PSAS scores were associated with greater perceived food responsiveness in the infant (*β* = 0.43; *p <* .001).

**Table 4:** Hierarchical regression analysis demonstrating postpartum specific anxiety as a predictor of infant food responsiveness after controlling for general measures of mood

Food responsiveness	Cumulative		Simultaneous	
	*R* ^2^-change	*F*-change	*Β*	*p*
Step 1				
Infant age	.12	*F* (4,255) = 8.97**	*− 0.13*	*.02*
Birth order			*0.18*	*.003*
Any BF activity			*0.30*	*<.001*
Anxiety/depression diagnosis			0.01	.89
Step 2				
BDI	.09	*F* (3,252) = 9.87**	0.08	.52
STAI-S			− 0.17	.17
STAI-T			0.08	.54
Step 3				
PSAS	.06	*F* (1,251) = 19.43**	*0.43*	*<.001*

Also after controlling for covariates identified as significant confounders in bivariate analyses in step 1. Italic entries indicate significant *β* and *p* values

*BF* breastfeeding

** *p* < .001

### HMR predicting infant satiety responsiveness (Table 5)

The final regression model predicted approximately 10% of the variance in satiety responsiveness scores (*R* ^2^ = 0.10, *F* (8,251) = 3.65, *p* < .001). The covariates in step 1 explained approximately 7% of the variance. General anxiety and depression (step 2) explained approximately 2% of the variance in scores; only general depression was significant and negatively associated with the outcome (*β* = − 0.29, *p =* .047). In the final step, the PSAS was also a significant predictor which explained approximately 6% of the variance in satiety responsiveness scores. However, PSAS scores were positively associated with perceptions of satiety responsiveness (*β* =  .24; *p =* .03).

**Table 5:** Hierarchical regression analysis demonstrating postpartum-specific anxiety as a predictor of infant satiety responsiveness after controlling for general measures of mood

Satiety responsiveness	Cumulative		Simultaneous	
	*R* ^2^-change	*F-*change	*β*	*p*
Step 1				
UK/non-UK	.07	*F* (4,255) = 4.94**	*− 0.13*	*.03*
Birth order			*0.17*	*.01*
Any BF activity			*− 0.15*	*.01*
Anxiety/depression diagnosis			0.10	.17
Step 2				
BDI	.02	*F* (3,252) = 1.42	*− 0.29*	*.047*
STAI-S			0.12	.38
STAI-T			0.03	.84
Step 3				
PSAS	.06	*F* (1,251) = 4.71*	*0.24*	*.03*

Also after controlling for covariates identified as significant confounders in bivariate analyses in step 1. Italic entries indicate significant *β* and *p* values

*BF* breastfeeding

*p < .05

**p < .001

### HMR predicting infant slowness in eating (Table 6)

The final regression model predicted approximately 8% of the variance in slowness of eating scores (*R* ^2^ = .08, *F* (7,252) = 3.01, *p* = .005). The covariates in step 1 explained approximately 6% of the variance. General anxiety and depression (step 2) explained approximately 1% of the variance in scores and were not significant. In the final step, the PSAS explained approximately 1% of the variance and was not a significant predictor of slowness in eating.

**Table 6:** Hierarchical regression analysis demonstrating postpartum specific anxiety as a predictor of infant slowness in eating after controlling for general measures of mood

Slowness in Eating	Cumulative		Simultaneous	
	*R* ^2^-change	*F*-change	*β*	*p*
Step 1				
Infant age	.06	*F* (3,256) = 4.94*	*− 0.13*	*.03*
Timing of ICF			*0.06*	*.01*
Anxiety/depression diagnosis			0.07	.33
Step 2				
BDI	.01	*F* (3,253) = 1.24	0.18	.20
STAI-S			− 0.17	.22
STAI-T			− 0.05	.73
Step 3				
PSAS	.01	*F* (1,252) = 2.40	0.16	.12

Also after controlling for covariates identified as significant confounders in bivariate analyses in step 1. Entries in italic indicate significant *β* and *p* values

*BF* breastfeeding, *ICF* introduction to complementary feeding

*p < .05

### HMR predicting infant general appetite (Table 7)

The final regression model predicted approximately 10% of the variance in general appetite scores (*R* ^2^ = .10, *F* (8,251) = 3.27, *p* = .001). The covariates in step 1 explained approximately 9% of the variance in general appetite. General anxiety and depression scores (step 2) explained approximately 1% of the variance, although these predictors were not significant. In the final step, PSAS scores explained no variance and were not a significant predictor of general appetite.

**Table 7:** Hierarchical regression analysis showing postpartum specific anxiety as a predictor of infant general appetite after controlling for general measures of mood

General appetite	Cumulative		Simultaneous	
	*R* ^2^-change	*F*-change	*β*	*p*
Step 1				
Infant age	.09	*F* (4,255) = 6.24*	*− 0.18*	*.003*
Timing of ICF			*− 0.15*	*.02*
Birth weight (g)			*0.13*	*.03*
Any BF activity			*0.17*	*.008*
Step 2				
BDI	.01	*F* (3,252) = 0.49	0.14	.30
STAI-S			− 0.04	.76
STAI-T			− 0.03	.81
Step 3				
PSAS	.000	*F* (1,251) = 0.02	− 0.03	.88

Also after controlling for covariates identified as significant confounders in bivariate analyses in step 1. Italic entries indicate significant *β* and *p* values

*BF* breastfeeding, *ICF* introduction to complementary feeding

*p < .001

## Discussion

Results demonstrate that higher levels of postpartum-specific anxiety are associated with lower odds of breastfeeding exclusively, and breastfeeding in any quantity in the first 6 months postpartum. These findings provide evidence for the predictive validity of the PSAS as they are comparable with a body of literature which finds that PPA is inversely associated with exclusive breastfeeding (Virden 1988; Groër 2005; Clifford 2006; Britton 2007; Zanardo et al. 2009; Adedinsewo et al. 2014) and breastfeeding in any quantity (Adedinsewo et al. 2014; Britton 2007; Brown and Arnott 2014; Courtois et al. 2014; Paul et al. 2013). Biological theories of anxiety and lactation posit that PPA may negatively influence breastfeeding through physiological stress responses and subsequent hormone imbalance (Lonstein 2007). In particular, anxiety is associated with lower oxytocin and prolactin which may inhibit the milk ejection reflex and subsequent breast milk production (Chen et al. 1998; Dewey 2001; Stuebe et al. 2012). It is theorised that postpartum-specific anxieties negatively affect breastfeeding practices via the same physiological mechanisms.

The current study also observed that postpartum-specific anxiety was significantly associated with negative perceptions of infant-feeding behaviours including a lower enjoyment of food, and greater food responsiveness and satiety responsiveness in the infant. The latter two findings may initially appear counterintuitive; food responsiveness is characterised by excessive hungriness, unnecessary and frequent demands for milk, and an inability to recognise satiety cues. Conversely, satiety responsiveness is characterised by under-consumption of milk during feeds and an oversensitivity to cues of satiety (Llewellyn et al. 2011). However, all three findings map onto previous work which finds high levels of anxiety negatively affect maternal perceptions of infant-feeding behaviour and impede maternal ability to interpret infant cues of hunger and satiety (Farrow and Blissett 2005; Hellin and Waller 1992; Hurley et al. 2008; Richter and Reck 2013; Savage et al. 2008). This study adds to this by demonstrating that postpartum-specific anxiety can negatively affect maternal perceptions across a number of feeding domains simultaneously and regardless of polarity. Distorted perceptions are a fundamental characteristic of unresponsive feeding practices which are linked to a range of adverse health outcomes (Cullen et al. 2000; Farrow and Blissett 2008; Hurley et al. 2011). Despite these findings, postpartum-specific anxiety was not associated with slowness in eating or general appetite. Given that this was the case for all of the measures of mood in these models it may be that these domains of feeding behaviour are too diffuse or that they do not elicit the same emotional response from mothers.

As hypothesised, postpartum-specific anxiety was a stronger predictor of infant-feeding outcomes and behaviours than general anxiety (state and trait) and depression. The PSAS was the only significant measure of mood across all of the feeding outcomes and behaviours apart from slowness in eating and general appetite analyses (in which none of the measures of mood were significant). Furthermore, the PSAS was a significant predictor after controlling for general anxiety and depression indicating that postpartum-specific anxiety elicits *a unique effect* upon infant-feeding outcomes and behaviours. This is a novel finding in the postpartum anxiety literature and resonates with a body of work which finds pregnancy-specific anxiety is a more potent predictor across a range of infant health and behaviour outcomes (Davis and Sandman 2010; Dunkel Schetter 2011; Guardino and Schetter 2014; Huizink et al. 2002, 2003). Theories of pregnancy-specific anxiety posit that it is a distinct construct which is rooted in the emotional and physical context of a specific pregnancy (Huizink et al. 2004). It has been suggested that pregnancy-specific anxiety may differ in its predictive power because it is more proximally linked to physiological stress responses than general measures of mood (Guardino and Schetter 2014). Furthermore, studies attempting to distinguish between general and pregnancy-specific anxiety typically report moderate correlations, suggesting that there is overlap but there is also an inimitable construct (Rini et al. 1999; Green et al. 2003; Huizink et al. 2004). These findings extend the applicability of this theory to the postpartum period. It is proposed that postpartum-specific anxiety is a distinct construct which is embedded in the emotional and physical context of the months following childbirth with a new infant. The study provides new evidence for childbearing specific measures of mood in the postnatal period and calls for an increased uptake in the use of these measures when attempting to predict childbearing related outcomes. Future research should aim to replicate these findings across other indices of maternal and infant health and behaviour in the postpartum period, particularly those with previously inconsistent results using general measures.

One strength of this study is its simultaneous consideration of infant-feeding outcomes and behaviours which provides a more comprehensive overview of the relationship between PPA and infant-feeding than other work (Fallon et al. 2016a). Furthermore, the analysis distinguished between exclusive and any breastfeeding which is consistent with current breastfeeding recommendations (McAndrew et al. 2012) and more detailed than previous research (Dusdieker et al. 1985; Hellin and Waller 1992; Mezzacappa and Katkin 2002; Cooke et al. 2007; O’Brien et al. 2008; Courtois et al. 2014). However, some limitations must also be acknowledged. Although the study controlled for a range of established confounders, the short-term prospective design precludes causality. Future research should aim to replicate the findings prospectively over a longer follow-up period. An online convenience sample was used which was adequately powered for the analyses conducted but lacked sampling control. As such, the samples were predominately married, primiparous, housewives which limit the generalizability of findings to other populations. Finally, although the PSAS was a highly significant predictor in the models discussed, the variance explained in the outcome variables was low which indicates that there is a reliable, albeit small, relationship between variables. Infant-feeding practices are complex and multifaceted, with many cultural (Scott et al. 2015), social (Hauff 2014), physical (Arbour and Kessler 2013), and emotional (O’Brien et al. 2008) factors affecting behaviours and outcomes. Given the current lack of uptake to infant-feeding recommendations (McAndrew et al. 2012), identification of any factor that consistently impacts upon feeding practices is important.

The domains of anxiety encompassed in the PSAS may all be potentially modifiable through support, education, and treatment. Replication of these findings in relation to infant-feeding and other fundamental maternal and infant health outcomes will provide an evidence base to inform interventions aimed at reducing postpartum-specific anxiety. Interventions designed to alter feeding perceptions in anxious postpartum populations may also increase the likelihood of positive feeding interactions, reduce the onset of feeding difficulties, and alleviate the emotional consequences brought about by them.

## References

[CR1] Adedinsewo DA, Fleming AS, Steiner M (2014). Maternal anxiety and breastfeeding: findings from the MAVAN (Maternal Adversity, Vulnerability and Neurodevelopment) study. J Hum Lact.

[CR2] Arbour MW, Kessler JL (2013). Mammary hypoplasia: not every breast can produce sufficient milk. J Midwifery Womens Health.

[CR3] Beck AT, Steer RA, Carbin MG (1988). Psychometric properties of the Beck Depression Inventory: twenty-five years of evaluation. Clin Psychol Rev.

[CR4] Birch L, Fisher J (1995). Appetite and eating behavior in children. Pediatr Clin N Am.

[CR5] Britton JR (2007). Postpartum anxiety and breastfeeding. J Reprod Med.

[CR6] Brown A, Arnott B (2014). Breastfeeding duration and early parenting behaviour: the importance of an infant-led, responsive style. PLoS One.

[CR7] Chen DC, Nommsen-rivers L, Dewey KG, Lönnerdal B (1998). Stress during labor and delivery and early lactation performance. Am J Clin Nutr.

[CR8] Clifford TJ (2006). Factors influencing full breastfeeding in a southwestern Ontario community: assessments at 1 week and at 6 months postpartum. J Hum Lact.

[CR9] Cooke M, Schmied V, Sheehan A (2007). An exploration of the relationship between postnatal distress and maternal role attainment, breast feeding problems and breast feeding cessation in Australia. Midwifery.

[CR10] Courtois E, Lacombe M, Tyzio S (2014) Facteurs associ{é}s a la poursuite de l’allaitement jusqu’{à} 6 mois chez les m{è}res allaitantes dans une maternit{é} parisienne. Rech Soins Infirm 50–64. doi:10.3917/rsi.117.0050 25080624

[CR11] Cullen KW, Baranowski T, Rittenberry L (2000). Socioenvironmental influences on children’s fruit, juice and vegetable consumption as reported by parents : reliability and validity of measures. Public Health Nutr.

[CR12] Davis EP, Sandman CA (2010). The timing of prenatal exposure to maternal cortisol and psychosocial stress is associated with human infant cognitive development. Child Dev.

[CR13] Davis EP, Snidman N, Wadhwa PD (2004). Prenatal maternal anxiety and depression predict negative behavioral reactivity in infancy. Infancy.

[CR14] Dewey KG (2001). Maternal and fetal stress are associated with impaired lactogenesis in humans. J Nutr.

[CR15] Dunkel Schetter C (2011). Psychological science on pregnancy: stress processes, biopsychosocial models, and emerging research issues. Annu Rev Psychol.

[CR16] Dusdieker LB, Booth BM, Seals BF, Ekwo EE (1985). Investigation of a model for the initiation of breastfeeding in Primigravida women. Soc Sci Med.

[CR17] Fairlee T, Gillman MW, Rich-edwards J (2009). High pregnancy-related anxiety and prenatal depressive symptoms as predictors of intention to breastfeed and breastfeeding initiation. J Womens Health.

[CR18] Fallon V, Groves R, Halford JCG (2016). Postpartum anxiety and infant-feeding outcomes: a systematic review. J Hum Lact.

[CR19] Fallon V, Halford JCG, Bennett KM, Harrold JA (2016). The postpartum specific anxiety scale: development and preliminary validation. Arch Womens Ment Health.

[CR20] Farrow CV, Blissett JM (2005). Is maternal psychopathology related to obesigenic feeding practices at 1 year?. Obes Res.

[CR21] Farrow CV, Blissett J (2008). Controlling feeding practices: cause or consequence of early child weight. Pediatrics.

[CR22] Glasheen C, Richardson GA, Fabio A (2010). A systematic review of the effects of postnatal maternal anxiety on children. Arch Womens Ment Health.

[CR23] Green J, Kafetsios K, Statham H, Snowdon C (2003). Factor structure, validity and reliability of the Cambridge worry scale in a pregnant population. J Health Psychol.

[CR24] Groër MW (2005). Differences between exclusive breastfeeders, formula-feeders, and controls: a study of stress, mood, and endocrine variables. Biol Res Nurs.

[CR25] Guardino CM, Schetter CD (2014). Understanding pregnancy anxiety: concepts, correlates and consequences. Zero Three.

[CR26] Hauff L (2014). Associations of maternal obesity and psychosocial factors with breastfeeding intention, initiation, and duration. Am J Clin Nutr.

[CR27] Hellin K, Waller G (1992). Mothers’ mood and infant feeding: prediction of problems and practices. J Reprod Infant Psychol.

[CR28] Hughes SO, Power TG, Orlet Fisher J (2005). Revisiting a neglected construct: parenting styles in a child-feeding context. Appetite.

[CR29] Huizink AC, Robles De Medina PG, Mulder EJH (2002). Psychological measures of prenatal stress as predictors of infant temperament. J Am Acad Child Adolesc Psychiatry.

[CR30] Huizink AC, Robles de Medina PG, Mulder EJH (2003). Stress during pregnancy is associated with developmental outcome in infancy. J Child Psychol Psychiatry.

[CR31] Huizink AC, Mulder EJH, Robles de Medina PG (2004). Is pregnancy anxiety a distinctive syndrome?. Early Hum Dev.

[CR32] Hurley KM, Black MM, Papas MA, Caulfield LE (2008). Maternal symptoms of stress, depression, and anxiety are related to nonresponsive feeding styles in a statewide sample of WIC participants. J Nutr.

[CR33] Hurley KM, Cross MB, Hughes SO (2011). A systematic review of responsive feeding and child obesity in high-income countries. J Nutr.

[CR34] Levin JS (1991). The factor structure of the pregnancy anxiety scale. J Health Soc Behav.

[CR35] Llewellyn CH, van Jaarsveld CHM, Johnson L (2011). Development and factor structure of the Baby Eating Behaviour Questionnaire in the Gemini birth cohort. Appetite.

[CR36] Lonstein JS (2007). Regulation of anxiety during the postpartum period. Front Neuroendocrinol.

[CR37] Mcandrew AF, Thompson J, Fellows L et al (2012) Infant feeding survey 2010. Heal Soc Care Inf Cent 1–331

[CR38] Meades R, Ayers S (2011). Anxiety measures validated in perinatal populations: a systematic review. J Affect Disord.

[CR39] Mezzacappa ES, Katkin ES (2002). Breast-feeding is associated with reduced perceived stress and negative mood in mothers. Health Psychol.

[CR40] O’Brien M, Buikstra E, Hegney D (2008). The influence of psychological factors on breastfeeding duration. J Adv Nurs.

[CR41] Paul IM, Downs DS, Schaefer EW (2013). Postpartum anxiety and maternal-infant health outcomes. Pediatrics.

[CR42] Richter N, Reck C (2013). Positive maternal interaction behavior moderates the relation between maternal anxiety and infant regulatory problems. Infant Behav Dev.

[CR43] Rini CK, Dunkel-Schetter C, Wadhwa PD, Sandman CA (1999). Psychological adaptation and birth outcomes: the role of personal resources, stress, and sociocultural context in pregnancy. Health Psychol.

[CR44] Savage JS, Fisher JO, Birch LL (2008). Parental influence on eating behavior. NIH Public Access.

[CR45] Scott JA, Kwok YY, Synnott K (2015). A comparison of maternal attitudes to breastfeeding in public and the association with breastfeeding duration in four European countries: results of a cohort study. Birth.

[CR46] Smith T, Kipnis G (2012). Implementing a perinatal mood and anxiety disorders program. MCN Am J Matern Nurs.

[CR47] Spielberger C, Gorsuch R, Lushene R (1970). Manual for the state-trait anxiety inventory.

[CR48] Stuebe AM, Grewen K, Pedersen CA (2012). Failed lactation and perinatal depression: common problems with shared neuroendocrine mechanisms?. J Women's Health (Larchmt).

[CR49] Van den Bergh B (1990). The influence of maternal emotions during pregnancy on fetal and neonatal behavior. J Prenat Perinat Psychol Health.

[CR50] Virden SF (1988). The relationship between infant feeding method and maternal role adjustment. J Nurse Midwifery.

[CR51] Wadwha PD, Sandman CA, Porto M (1993). The association between prenatal stress and infant birth weight and gestational age at birth: a prospective investigation. Am J Obstet Gynecol.

[CR52] WHO (2002) Indicators for assessing infant and young child feeding practices. Retrieved from http://apps.who.int/iris/bitstream/10665/43895/1/9789241596664_eng.pdf

[CR53] Zanardo V, Gasparetto S, Giustardi A (2009). Impact of anxiety in the puerperium on breast-feeding outcomes: role of parity. J Pediatr Gastroenterol Nutr.

